# Imaging Tumor Vascularity and Response to Anti-Angiogenic Therapy Using Gaussia Luciferase

**DOI:** 10.1038/srep26353

**Published:** 2016-05-20

**Authors:** Rami S. Kantar, Ghazal Lashgari, Elie I. Tabet, Grant K. Lewandrowski, Litia A. Carvalho, Bakhos A. Tannous

**Affiliations:** 1 Experimental Therapeutics and Molecular Imaging Laboratory, Neuroscience Center, Department of Neurology, Massachusetts General Hospital, Boston, Massachusetts, USA; 2 Program in Neuroscience, Harvard Medical School, Boston, Massachusetts, USA

## Abstract

We developed a novel approach to assess tumor vascularity using recombinant *Gaussia* luciferase (rGluc) protein and bioluminescence imaging. Upon intravenous injection of rGluc followed by its substrate coelenterazine, non-invasive visualization of tumor vascularity by bioluminescence imaging was possible. We applied this method for longitudinal monitoring of tumor vascularity in response to the anti-angiogenic drug tivozanib. This simple and sensitive method could be extended to image blood vessels/vasculature in many different fields.

Tumorigenesis is associated with increased vascularity to maintain adequate supplies of oxygen and nutrients^1^,^2^,^3^. Several anti-angiogenic therapies targeting tumor vascular moieties such as vascular endothelial growth factor (VEGF) receptor as well as integrins, for several tumor types have been established as a promising therapeutic approach^4^,^5^,^6^. These therapies appear to be more cytostatic rather than cytotoxic, and therefore, are not desirably assessable with traditional imaging modalities that evaluate tumor volume^7^. As a consequence, adequate methods of assessing tumor vascularity and the response to anti-angiogenic therapeutics remain controversial^8^. Recent advancements in molecular imaging techniques have provided the possibility of noninvasive assessment of tumor response to different therapies. Several reports using MicroPET/CT imaging with different radiolabeled tracers to monitor anti-angiogenic therapies have been described^9^,^10^. These tracers mostly target receptors on endothelial cells such as integrins. However, not all tumor types have predominantly high expression of integrins on endothelial cells, compared to tumor cells. For instance, melanoma tumors have been shown to have higher expression of a specific subset of integrins targeted with a tracer, as compared to the tumor vasculature^11^. In case where radiotracers are conjugated with an intact antibody, clearance from blood is relatively slow leading to a high background accumulation^9^. Another potential limitation of this method is the distinction between tumors and inflammation might not be feasible due to similar intense uptake by both inflammatory lesions and malignancies^12^. Fluorescence-based optical imaging methods have also been used to visualize tumor blood vessels. In this approach, vascular-specific fluorescent probes are synthesized by conjugating antibodies such as anti-VEGF antibody with a fluorescent dye^13^,^14^. In contrast to microPET, fluorescence imaging does not require radioactive materials, and minimal tissue autofluorescence allows efficient photon penetration and subsequently enhanced target-to-background ratios^14^. More recently, Fab fragment of antibodies are used instead of intact antibodies to overcome the long half-life of high molecular weight, leading to accumulation in the liver^15^. One limitation for using fluorescence imaging is that accurate quantification of the results is challenging. Another caveat is the depth of the tissue that can be evaluated which restricts its clinical application to only superficial tumors^16^. This approach also relies on sophisticated and expensive instrumentation including multiphoton microscopy, fluorescence tomography and intravital imaging^17^,^18^. In addition to these techniques, different MRI parameters such as contrast enhancement have been introduced as potential angiogenesis biomarkers^19^. Most of these parameters were found to be reflection of the physiological changes in tumor vascularity such as perfusion and permeability, rather than accurate measures of microvascular density. Therefore, contrast-enhanced MRI is more often used to assess the structural and hemodynamic status of solid tumors^7^. Vessel-caliber MRI on the other hand has emerged over the past decades as a potential method to monitor anti-angiogenic therapy in clinical trials^20^,^21^. This approach takes advantage of the formation of abnormal tumor vessels with a wide spectrum of calibers in cancer tissue and quantifies the average vessel diameters and average vessel densities for arteries, capillaries and veins^7^. Although a correlation between tumor vascular status, tumor grade and response to therapies has been found in several studies^22^,^23^,^24^, the complex process of image acquisition in vessel-caliber MRI justifies the limited attention this technique has received^25^.

Bioluminescence imaging on the other hand has the advantage of simplicity with negligible background signal^26^,^27^. It relies on production of light, following a chemical reaction in which the enzyme (luciferase) oxidizes a substrate leading to photon emission. Typically, cells of interest are engineered *ex vivo* to express the luciferase reporter under the control of a constitutive or tissue/process-specific promoter, then implanted into the animal and tracked non-invasively upon injection of the corresponding substrate^26^,^28^,^29^,^30^. Recently, we and others have shown that the naturally secreted *Gaussia* luciferase (Gluc) could be used for quantitative assessment of different biological processes in mice by measuring its level in microliters of blood *ex vivo*^28^,^31^,^32^,^33^,^34^. Gluc is the smallest luciferase cloned (19.9 kDa) and is thermostable, making it very suitable for different applications. Given the advantages of this luciferase, we evaluated here the potential use of recombinant Gluc protein (rGluc) in combination with its substrate coelentrazine, for noninvasive and real-time imaging of tumor vascularity in small animals.

## Results and Discussion

**Figure 1:**
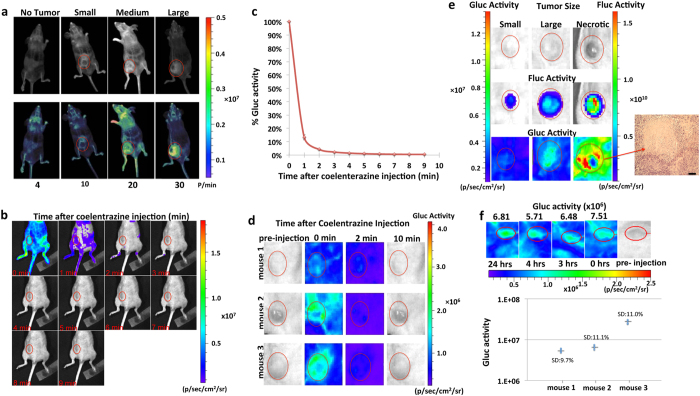
Optimization of rGluc assay to image tumor vascularity. (**a**) Increasing number of U87 cells or PBS (control) were implanted in nude mice (*n* = 4). Two weeks later, mice were injected with rGluc followed by coelenterazine and imaged using a CCD camera. Gluc signal in photons/min (p/min) for different tumor sizes is indicated. Overlay of light with bioluminescence (top) or pseudocolor (bottom) images are shown. (**b–d**) Tumor-bearing mice (n = 3) were injected with rGluc followed by coelenterazine retro-orbitally and images were acquired every minute over 10 minutes. Average % of total photon flux (where first minute is set at 100%) ± SD is plotted (**b**). Bioluminescence imaging from a representative mouse with tumor-associated signal at every time point is shown (**c**). Bioluminescent images of mice with different tumor sizes are shown at different time points (**d**). (**e**) Gluc-based bioluminescence imaging of tumor vascularity as well as Fluc imaging (for tumor volume) was performed for small, large and necrotic tumors; showing an H&E staining of the necrotic tumor; scale bar 200 μm. (**f**) Repeated imaging of the same mice at four different time points over 24 hrs with standard deviation (SD) presented for each mouse (n = 3). Representative bioluminescence images from mouse 2 are shown in the upper panel.

We first designed an *in vivo* rGluc-based bioluminescence assay for non-invasive imaging of tumor vascularity. We implanted different numbers of U87 human glioblastoma cells subcutaneously into the flanks of nude mice. Two weeks post-implantation, when tumors reached different sizes, we visualized tumor vascularity by *in vivo* bioluminescence imaging after intravenous (i.v) injection of recombinant Gluc (rGluc; 10 mg/kg body weight) followed by its substrate coelenterazine (5 mg/kg body weight) and acquisition of photon counts for 1 minute using a cooled charge-coupled device (CCD) camera. The captured signal intensity positively correlated with tumor size, number of implanted cells, and clear visualization of tumor vascularity (Fig. 1a). Furthermore, we could visualize all mice blood vessels, especially in control animals with no tumors (Fig. 1a). We optimized our method by performing kinetics analysis and observed that immediate imaging post-coelenterazine injection (1 min post-rGluc injection with signal acquisition for 1 min) gave the highest signal reaching background level within 10 minutes (Fig. 1b–d) similar to our published work^26^,^28^,^29^,^30^. Interestingly, no signal was detected after 10 minutes regardless of the tumor size (Fig. 1d). This indicates a fast assay turnover, allowing dynamic longitudinal monitoring of tumor vascularity at different time points without residual signal accumulation, concordant with the fast systemic clearance of Gluc (half-life <20 min)^28^. Further, necrotic tumors had a strong signal at their periphery and a weak to absent signal at their center (Fig. 1e). These results confirm that signals obtained within and around the tumor indeed reflect vascularity, since necrotic tumors are known to have numerous vessels at their growing rim and fewer feeding vessels in their core^35^. Finally, we assessed the reproducibility of our assay by repeated imaging of the same tumor-bearing mice and observed similar signal with <12% standard deviation among four different time points over 24 hrs (Fig. 1f).

**Figure 2:**
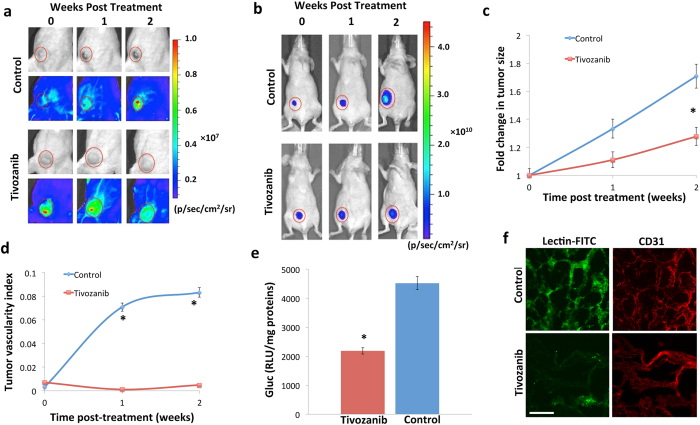
Bioluminescence imaging of tumor vascularity and response to anti-angiogenic therapy. Mice were subcutaneously implanted with 10^6^ U87-Fluc cells and one week later, randomized and treated with either vehicle (control) or tivozanib daily for 2 weeks (*n* = 6/group). (**a**) Mice were imaged for tumor vascularity after injection of rGluc followed by coelenterazine. (**b**) Tumor volume was monitored after injection of D-luciferin, the Fluc substrate. A representative mouse from each group is shown. (**c**) Tumor size was measured weekly using a manual caliper and average fold changes in tumor size was calculated. (**d**) Tumor vascularity index (ratio of tumor rGluc signal to Fluc signal) was calculated at different time points. Results are represented as mean ± SD (*P < 0.05). (**e**) *Ex vivo* rGluc activity was analyzed in tumor homogenates using a luminometer after addition of coelenterazine. Results are represented as mean of rGluc activity in relative luminescence units (RLU) in tumor homogenates (normalized to total protein) ± S.D (*P = 0.03). (**f**) Mice from both groups were injected with Fluorescein-lectin and tumors were excised, sectioned and evaluated for Fluorescein (vascular endothelium) or immunostained with anti-CD31 antibody and analyzed by fluorescence microscopy. Scale bar, 50 μm.

We then explored if our method could assess tumor vasculature response to anti-angiogenic therapies. We engineered U87 cells to stably express firefly luciferase (U87-Fluc)^28^. Fluc imaging here allows concomitant tumor size quantification with rGluc imaging of tumor vascularity. We implanted one million U87-Fluc cells subcutaneously into the flanks of nude mice and once tumors were formed (1 week post-implantation), mice were randomized into two groups; one group received oral anti-angiogenic therapy (1 mg/kg of tivozanib in DMSO/0.5% methylcellulose) while the control group received the drug vehicle (DMSO in 0.5% methylcellulose in water) daily for two weeks. We then applied our assay to evaluate tumor vascularity before and at different time-points post-treatment, by injecting rGluc (10 mg/kg body weight) followed by coelenterazine (5 mg/kg body weight) and imaging immediately over 1 minute. We observed a decrease in tumor-associated rGluc signal (vascularity) in the tivozanib-treated group, while it increased in control mice (Fig. 2a). To correlate these findings with tumor size, we concomitantly imaged the implanted tumors using Fluc-based bioluminescence imaging, which showed slower growth in the treated mice (Fig. 2b). These results were validated by weekly manual caliper measurements of tumor size in both groups (Fig. 2c). Since larger tumors are expected to have a richer vascular supply, we applied a tumor vascularity index to standardize the observed tumor vascularity to tumor size (rGluc/Fluc signal ratio). Calculation of the tumor vascularity index showed a significant (p < 0.05) decrease in the tivozanib-treated mice over time (Fig. 2d).

We further validated our results with additional *ex vivo* experiments. Two weeks post-treatment, treated and control mice were injected with rGluc and one-minute later fresh tumors were excised, homogenized and analyzed for rGluc activity *ex vivo* using a luminometer. Consistent with our *in vivo* data, rGluc activity (normalized to total protein) was significantly (almost two folds; P = 0.03) decreased in the tivozanib-treated tumor homogenates (Fig. 2e). To further confirm these results, mice were injected with fluorescein lycopersicon esculentum (tomato) lectin (5 mg/kg body weight), an effective vascular endothelium marker in rodents. Mice were then perfused and tumors were resected, sectioned and analyzed by fluorescence microscopy. We observed that control tumor sections had a stronger fluorescein signal (therefore increased vascularity) as compared to tivozanib-treated tumors (Fig. 2f). We also stained these tumor sections for CD31, a known vessel-specific marker, and also observed a decrease in CD31 positivity in the tivozanib-treated group (Fig. 2f). All of these data demonstrate that systemically-injected rGluc indeed distribute in the tumor vasculature and can be used as a reporter to image tumor vascularity and its response to anti-angiogenic therapy, noninvasively and in real-time.

**Figure 3:**
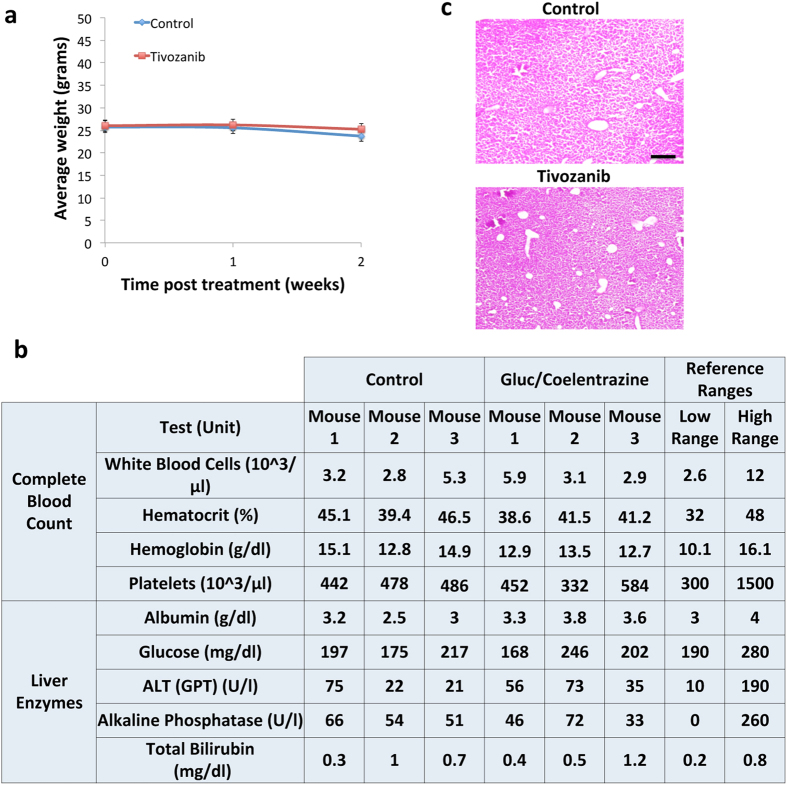
Toxicity analysis of rGluc tumor vascularity assay. (**a**) Mice weight from control and tivozanib-treated groups was monitored weekly. (**b,c**) Mice were injected with rGluc followed by coelenterazine for 2 weeks (3x/week) or PBS (control; n = 3/group). Blood samples were collected from both groups and analyzed for complete blood count and liver enzymes (**b**). Livers were collected, sectioned, and stained for Hematoxylin and Eosin (**c**). Shown is a representative liver section staining from each group. Scale bar, 20 μm.

Finally, we evaluated potential cytotoxicity of repetitive injection of rGluc and coelenterazine. We weighed all mice weekly throughout the treatment duration and did not observe any changes in their weight (Fig. 3a). We also collected blood samples from both groups and analyzed them for liver enzymes and complete blood counts and again did not observe any major differences (Fig. 3b). Furthermore, Hematoxylin and Eosin staining on liver sections from both groups did not show any signs of toxicity (Fig. 3c). All together, these results suggest that tumor vascularity imaging using rGluc/coelenterazine does not lead to any toxicity in mice.

## Conclusion

In this study, we describe a novel bioluminescent rGluc-based assay for non-invasive imaging and longitudinal monitoring of tumor vascularity and its response to anti-angiogenic therapy. To our knowledge, this is the first described method to use recombinant luciferase and bioluminescence imaging for this purpose. This method provides a simple, fast, sensitive, safe and cost-effective way to image tumor vascularity, response to therapeutics, and could be applied to different fields and diseases involving vascular processes.

## Methods

### Expression vectors

The firefly luciferase was previously cloned into a lentivirus vector under the control of the strong constitutive cytomegalovirus (CMV) promoter^28^. Lentivirus vector stocks were produced by triple transfection of 293T cells (provided by Dr. Michele Calos, Stanford Univ. Sch. Med.) with the lentivirus vector plasmid, the packaging genome plasmid, pCMV∆R8.91, and the plasmid, pVSV-G (Clontech) encoding the vesicular stomatitis virus envelope glycoprotein as described^36^. Vector stocks were used to transduce U87 glioblastoma cells (obtained from ATCC) with 20 transducing units (TU)/cell to produce U87-Fluc.

### Tumor models

All animal studies were approved by the Subcommittee on Research Animal Care at the Massachusetts General Hospital and were performed in accordance to their guidelines and regulations. Athymic female nude mice were anesthetized with intraperitoneal (i.p) injection of ketamine (100 mg/kg) and xylazine (5 mg/kg). The skin was cleaned by scrubbing with 70% ethanol pads, followed by scrubbing with betadine pads. Different number of U87-Fluc cells in 50 μl PBS were pre-mixed with an equal volume of Matrigel (BD Bioscience) and implanted into the flanks of these mice.

*In vivo* bioluminescence imaging. Mice were anesthetized with isoflurane (Baxter) and the rGluc tumor vascularity assay was performed using *in vivo* bioluminescence imaging as follows: rGluc (10 mg/kg body weight; a kind gift from Dr. Bruce Bryan, Nanolight) was i.v. injected retro-orbitally followed (1 minute later) by the Gluc substrate coelentrazine (5 mg/kg body weight; Nanolight) and immediate acquisition of photon counts for 1 minute using the IVIS Spectrum Imaging System (PerkinElmer). Fluc imaging was performed in a similar way, 10 minutes after intraperitoneal (i.p) injection of 150 μl of D-Luciferin (4 mg/kg body weight) and recording photon counts using the IVIS Spectrum Imaging System. A light image of the animal was taken in the chamber using dim polychromatic illumination. Following data acquisition, post-processing and visualization was performed using either CMIR-Image, a program developed by the Center for Molecular Imaging Research using image display and analysis suite developed in IDL (Research Systems Inc., Boulder, CO; for Fig. 1a) or IVIS Spectrum image analysis. Regions of interest were defined using an automatic intensity contour procedure to identify bioluminescence signals with intensities significantly greater than the background. The mean, standard deviation, and sum of the photon counts in these regions were calculated as a measurement of rGluc or Fluc activity. For visualization purposes, bioluminescence images were fused with the corresponding white light surface images in a transparent pseudocolor overlay, permitting correlation of areas of bioluminescent activity with anatomy.

### *Ex vivo* rGluc assay

Two weeks post-treatment, mice were injected with rGluc (10 mg/kg body weight) under deep anesthesia and one-minute later, tumors were removed and homogenized in 300 μl M-PER™ Mammalian Protein Extraction Reagent (Pierce) using a manual grinder. Total protein was quantified in the tumor homogenates using BSA standards. rGluc activity was then measured using the FlexStation 3 microplate reader (Molecular Devices) in 10 μl samples of tumor homogenates containing the same amount of total proteins by adding 90 μl coelentrazine (100 μM) in PBS-0.1% Triton X100 buffer.

### Tumor/liver staining

Two weeks post-treatment, mice were i.v. injected with Fluorescein Lycopersicon Esculentum (Tomato) Lectin (5 mg/kg body weight; Vector Laboratories). Animals were sacrificed by transcardial perfusion with phosphate buffered saline (PBS) followed by 4% paraformaldehyde (PFA), under deep anesthesia with intraperitoneal (i.p) injection of ketamine (100 mg/kg) and xylazine (5 mg/kg). Tumors were collected, soaked in 30% sucrose overnight and sectioned into 7 μm sections. Sections were mounted on slides and evaluated for Fluorescein staining using fluorescence microscopy. Similarly, livers were removed, sectioned and stained with Hematoxylin and Eosin and analyzed by microscopy.

For CD31 tumor staining, sections were washed with PBS and nonspecific binding was blocked with 5% normal goat serum +5% Rabbit serum +2% BSA (Bovine Serum Albumin) in PBS-T (PBS 1x + Triton 0.3%), for 1 hour at room temperature. Sections were then incubated overnight at 4 °C with anti-CD31 antibody (Abcam; 1:50 dilution) in 2% BSA/PBS. Slides were washed several times with PBS-T and then incubated with anti-rabbit IgG – 647 Alexa (1:700 dilution in 2%BSA/PBS) for 1 hour at room temperature. Slides were rinsed with PBS-T followed with 50 mM NH_4_Cl for 5 minutes to reduce the autofluorescence and then mounted on microscope slides with DAPI.

### Statistical analysis

Comparisons of data were performed using a two-tailed Student’s t test (unpaired) using Graphpad Prism 5. P values < 0.05 were considered significant.

## Additional Information

**How to cite this article**: Kantar, R. S. *et al*. Imaging Tumor Vascularity and Response to Anti-Angiogenic Therapy Using Gaussia Luciferase. *Sci. Rep.* **6**, 26353; doi: 10.1038/srep26353 (2016).
